# The Effects of a Cultivar and Silicon Treatments on Grain Parameters and Bioactive Compound Content in Organic Spring Wheat

**DOI:** 10.3390/foods14142406

**Published:** 2025-07-08

**Authors:** Iwona Kowalska, Mariusz Kowalczyk, Jarosław Mołdoch, Sylwia Pawelec, Paweł Radzikowski, Beata Feledyn-Szewczyk

**Affiliations:** 1 Department of Phytochemistry, Institute of Soil Science and Plant Cultivation-State Research Institute, Czartoryskich Str. 8, 24-100 Pulawy, Polandjmoldoch@iung.pulawy.pl (J.M.); spawelec@iung.pulawy.pl (S.P.); 2 Department of Agroecology and Economics, Institute of Soil Science and Plant Cultivation-State Research Institute, Czartoryskich Str. 8, 24-100 Pulawy, Poland; pradzikowski@iung.pulawy.pl (P.R.); bszewczyk@iung.pulawy.pl (B.F.-S.)

**Keywords:** silicon treatments, alkylresorcinols, LC-APCI-MS, phenolic acids, UPLC-DAD-MS, TLC-DPPH^•^, spring wheat grain

## Abstract

To address the need for improved nutritional value of organically grown wheat, this study investigated the impact of silicon treatments (AdeSil, ZumSil) on yield, health status, and bioactive compound content in spring wheat cultivars. The 2019–2020 research evaluated different application variants: seed dressing, foliar sprays, and their combinations. Comprehensive seed dressing combined with two foliar treatments, (variant B) and two foliar treatments (variant C), significantly increased yield (by an average of 8.9% and 7.6% vs. control, respectively). These variants beneficially affected fungal disease resistance mainly in the stressful 2019; in optimal 2020, they showed no clear advantage over the control, which performed similarly or better. Seed dressing (variant D) increased total phenolic acids (PAs) content and antioxidant activity, with the spelt cv. Wirtas exhibiting the highest levels. Silicon treatments modified alkylresorcinols (ARs) content, but effects depended on the year, cultivar, and application variant, not always exceeding the control. Silicon treatments, especially when applied in combination (seed dressing and foliar application), can improve spring wheat yield and favorably modify PAs content, enhancing grain nutritional value. However, the plant response regarding health status and ARs content is strongly conditioned by cultivar specificity and the prevailing environmental conditions of the growing year.

## 1. Introduction

Cereals represent a crucial raw material for the food industry, providing the basic ingredient for sustenance for approximately 40% of the global population [1]. Wheat grain is of pivotal importance as a strategic consumer commodity that influences global food security [2]. The factors that have been identified as contributing to this decline include changes in climatic conditions leading to biotic and abiotic stress, changes in farming practices due to economic or regulatory factors, and a decline in genetic progress resulting in stagnant yields. Consequently, it is imperative to establish the most efficacious solutions for growers to assist in the enhancement and advancement of cereal production. This objective can be realized through the adaptation of suitable cultivars, cultivation techniques, and formulations that promote optimal crop growth and quality [3].

In the context of the detrimental effects of synthetic fertilizers and plant protection products on soil ecology, a subject that has attracted significant attention in recent years, this issue is of considerable importance. Nutrient deficiencies arising from inadequate fertilization have resulted in diminished yields, reduced protein content, and increased vulnerability to diseases affecting cereal grains. This phenomenon is particularly evident in the case of wheat grain, which exhibits a high degree of susceptibility to fungal diseases [4,5].

Grain quality is the primary criterion for evaluating wheat on the global market, with its nutritional value impacting health not only through its nutrient content (e.g., protein, carbohydrates), but also through specific substances that exhibit radical-scavenging properties, such as phenolic acids and alkylresorcinols [6,7,8]. Research by Kowalska et al. [9,10] has demonstrated that the content of these substances in wheat grain varies between individual cultivars and depends on the cultivation system. Consequently, selecting suitable cultivars and implementing appropriate cultivation practices, which directly influence the content of these substances, will determine the nutritional value of wheat grain for consumers. The incorporation of wheat grain into the daily diet has the potential to significantly contribute to reducing numerous diet-related civilizational diseases [11,12]. Furthermore, the role of phenolic acids in enhancing the resistance of cereal grains to fungal pathogens has been demonstrated, with considerable implications for crop yield and quality, as well as consumer health and safety [13,14]. The production of toxins by fungal pathogens has been shown to pose a grave threat to consumer health [15]. In response to pathogens, phenolic acids induce the plant’s defense mechanism and synthesize compounds that initiate a specific hypersensitive reaction to prevent the spread of infection. However, in a stressful situation, when the plant is deprived of nutrients, it cannot support its immune system, devoting all its resources to basic life functions [14,16].

The ecological approach to plant production has had a significant impact on the development of work concerning substances of natural origin that could support the growth and health of crops, increase their resistance to abiotic stress (e.g., water deficiency and soil pollution), and enhance their ability to cope with such factors. The mechanism of plant resistance to these factors can be activated, among other things, by the plant signaling system, which is controlled by the supply of basic elements [17]. In this context, silicon (Si) merits particular consideration. Previous research has indicated that this element is particularly sensitive to intensive cultivation, particularly in monocultures of high-yield cultivars [18]. The polymerized form of Si naturally occurring in the soil is not absorbable by plants; however, it can be depolymerized through chemical processes in the soil and transported by the stem to the leaves. The final deposition of this compound in the leaf epidermis creates a protective layer against stress factors [19]. This process supports the use of silicon-containing treatments in a form capable of facile absorption by plants, thereby meeting contemporary demands for sustainable agriculture.

It has been demonstrated that the application of silicon biostimulants leads to enhancements the osmotic status of plants by minimizing water loss. This, in turn, establishes a physical barrier against pathogen attacks and ensures the undisturbed status of the physiological, biochemical, and molecular parameters of the plant. Furthermore, this process has been shown to stimulate antioxidant enzyme activity and inhibit the generation of active oxygen species, thus preventing toxicity [20].

Despite the demonstrable correlation observed between the utilization of silicon biostimulants and the increase of grain yield and biomass, accompanied by a diminution in losses attributable to pathogens, it was evident that the diversity of cultivar and the applied dose exerted a substantial influence [21,22]. However, there is a paucity of information regarding the impact of silicon biostimulants on the health-promoting properties of wheat grain. The present study aims to address this knowledge gap.

## 2. Materials and Methods

### 2.1. Characteristics of Sites, Agronomic Practices, and Design of Experiment

The field experiments were conducted in the years 2019–2020 at an Agricultural Experimental Station of the Institute of Soil Science and Plant Cultivation—State Research Institute (IUNG-PIB), in Grabów, Masovian Voivodeship, Poland [52°2′ N; 17°4′ E]. The experiment with the organic cultivation of spring wheat was set up on Cambisol soil in a split-block design with four replications. Each plot has an area of 30 m^2^. The previous crop was a legume-grass mixture. The experiment was a two-factor experiment, with four spring wheat cultivars as the first factor and the use of organic silicon biopreparations as the second factor. Organic silicon treatments: AdeSil (diatomaceous earth) and ZumSil (liquid organic silicon preparation), produced by Perma Guard Inc. (USA) (Table 1), were approved for use in organic farming and were used in the cultivation of spring wheat. Two silicon products were used: AdeSil as a solid formulation and ZumSil as a liquid trade formulation. AdeSil is an amorphous diatomaceous earth with a flour texture and contains 89–95% amorphous silica (SiO_2_), and ZumSil is a 24% solution of monosilicic acid. Different variants were used: A—untreated plot, B—seed dressing combined with two foliar treatments, C—two foliar treatments, and D—only seed dressing (Table 1).

Spring wheat was sown on the 10th and 8th of April of 2019 and 2020, respectively, with row spacing of 12.5 cm and a sowing rate of 500 grains per 1 m^2^ (Figure S1). No synthetic mineral fertilizers and pesticides were used. In 2019, spring wheat was harvested on 2 August, while in 2020, harvest was performed on 12 August.

### 2.2. Plant Material

The research material consisted of three cultivars of common wheat *Triticum aestivum* L. subsp. *aestivum* (cv. Harenda, Serenada, Rusałka) and one cultivar of spelt wheat *Triticum aestivum* L. subsp. *spelta* (cv. Wirtas). These spring wheat cultivars, listed in the National Register of Varieties, also differ in morphological characteristics and susceptibility to fungal pathogens, according to COBORU (www.coboru.gov.pl, accessed on 14 May 2025). The characteristics of common wheat cultivars are presented in the article of Kowalska et al. [23].

### 2.3. Grain Yield

The scope of the analyses included the assessment of the yield of spring wheat. Harvest was done using a special small harvester for the plot area of 25 m^2^ and then con-verted per t ha^−1^ at 15% moisture content. The yield and thousand-grain weight (TGW) were established in four replications.

### 2.4. Assessment of Plant Infestation by Pathogens

Fungal diseases of spring wheat were assessed in the first decade of July 2019 and 2020, determining the damage to the leaf surface caused by fungal pathogens: *Puccinia recondita*, *Septoria* sp., *Drechslera tritici-repentis*, *Blumeria graminis*, and *Fusarium* sp. Ten individual flag leaves were collected from each experimental plot. Leaves were collected at random locations within the plot, but always at least 1 m from its edge. The occurrence of symptoms of various fungal diseases was identified. Each detected symptom was assessed on a 0–100% scale, depending on the leaf surface area covered. The results presented average values from the assessment of leaf cover by fungal disease symptoms.

### 2.5. Meteorological Conditions

The meteorological conditions differed in the growing seasons of 2019 and 2020 (Table S1). In 2019, the temperature and precipitation were favorable for the emergence of spring wheat, which was even. The warm days in May contributed to a significant acceleration of the growth and development of the cereals. In June and July, the air temperature was favorable for the development and ripening of the plants, but there were periods of drought and heavy rainfall. This caused plant stress and led to fungal diseases, especially brown rust, at the end of the growing season. In 2020, the lack of rainfall from March to April was an unfavorable factor, but despite this, the emergence of spring wheat was even. In May, moderate rainfall and its good distribution ensured dynamic plant growth. In June, rainfall was abundant (157.8 mm). July was dry, which caused the plants to ripen faster and dry out.

### 2.6. Chemicals

Methanol and acetonitrile (>98%) were analytically graded and bought from J.T. Baker (Deventer, Netherlands). Chloroform, toluene, ammonium acetate, 2-propanol, 2,2-diphenyl-1-picrylhydrazyl radical (DPPH^•^), formic acid (LC-MS grade), and 5-*n*-heneicosylresorcinol were delivered by Sigma-Aldrich, (St. Louis, MO, USA). Acetone, n-hexane, and ethyl acetate were purchased from Acros Organics BVBA (Geel, Belgium). Other reagents (sodium hydroxide, ascorbic acid m-hydroxybenzoic acid, hydrochloric acid) were delivered by commercial suppliers, including Poland POCH S.A. (Gliwice, Poland) and Chempur (Piekary Śląskie). Milli-Q water was obtained using a purification system (Millipore Corp., Molsheim, France).

### 2.7. Analyses of Phenolic Acids (PAs)

#### 2.7.1. Extraction of PAs from Spring Wheat Cultivars

The ground material was degreased using a Soxhlet apparatus and n-hexane. Samples were prepared according to the modified method of Żuchowski et al. [24]. Hydrolysis was then performed by incubating the samples with 4M NaOH and 2% ascorbic acid for 4 h at room temperature. As an internal standard, 20 μg of m-hydroxybenzoic acid was added to each sample. After cooling on ice, the samples were acidified with 6M HCl to pH 2. The resulting solution was centrifuged (8000 rpm, 20 min), and the supernatant was extracted three times with ethyl acetate. After collecting and filtering the organic phase, the solvent was evaporated in a rotary evaporator (35 °C). The extract was dissolved in 30% methanol, transferred to Eppendorf tubes, and frozen (−20 °C).

#### 2.7.2. UPLC Analysis Conditions for PAs

Phenolic acids were determined using an ACQUITY UPLC system with a PDA detector and a triple quadrupole mass detector (TQD, Waters). Separation was performed on a Waters Acquity UPLC HSS C18 column (100 × 2.1 mm, 1.8 μm) at 30 °C. The mobile phase consisted of acidified water (0.1% formic acid) as solvent A and acidified acetonitrile (0.1% formic acid) as solvent B. Elution gradient: 8–20% B in 10.6 min, 20–95% B in 2.9 min, 95–8% B in 2 min. The sample injection volume was 2.5 μL, and the flow rate was 0.45 mL/min. Compound identification was based on UV and MS detection data. Data processing was performed using MassLynx V4.2 software (Waters) [6].

#### 2.7.3. Quantitative Determination of PAs

Phenolic acids were detected by multiple reaction monitoring (MRM) in negative ionization mode. The concentrations of the compounds (μg/g of the grain) were calculated based on calibration curves (Table S2).

#### 2.7.4. Antiradical Activity of PAs

The antioxidant activity of PAs in spring wheat cultivars was measured using a method previously reported by Kowalska et al. [25]. Briefly, the standard and phenolic fractions were plotted on silica TLC plates. Acetonitrile-water-chloroform-formic acid 60:15:10:5 (*v*/*v*/*v*/*v*) constituted the mobile phase. The plates were developed at 90 mm and dipped for 5 s in 0.2% (*w*/*v*) DPPH^•^ radical methanolic solution. Next, the plates were held in the dark for 30 min and scanned. The results of the TLC-DPPH^•^ test were stored in jpg documents and then converted by the ImageJ (1.48v; Java 1.6.0_20) program.

### 2.8. Analysis of Alkylresorcinols (ARs, Resorcinolic Lipids)

#### 2.8.1. Extraction of ARs from Wheat Samples

In order to estimate ARs contents, 1 g samples of powdered plant material were extracted with 40 mL of acetone using an ultrasonic bath at room temperature for 48 h. The extracts were then clarified by centrifugation at room temperature for 5 min (approximately 23,000× *g*), and the extracts were evaporated to dryness under reduced pressure. The extracts were then re-dissolved in 2 mL of acetonitrile, and 500 µL of each sample was filtered by 0.2 µm PTFE filter vials (Thomson) before LC-MS analysis.

#### 2.8.2. LC-APCI-MS Analysis of ARs

High-resolution LC-MS analyses were performed using a Thermo Scientific Ultimate 3000 RS chromatographic system connected to a Bruker Impact II HD quadrupole-time of flight (Q-TOF) mass spectrometer (Bruker, Billerica, MA, USA). The chromatographic separations were done on a Waters CORTECS C8 column (2.1 × 100 mm, 2.5 µm, Milford, CT, USA). Mobile phase A consisted of distilled water with 0.1% (*v*/*v*) formic acid and 1% (*v*/*v*) 1 M ammonium acetate, while mobile phase B was a mixture of 2-propanol and acetonitrile (3:7), also containing 0.1% (*v*/*v*) formic acid and 10 mM ammonium acetate. The elution was performed with isocratic elution from 0 to 0.5 min with 65% B, transitioning to a linear gradient from 0.5 to 20 min, reaching 98% B. The flow rate was maintained at 0.5 mL/min, and the column was kept at 35 °C. Between injections, the column was washed for 5 min with 100% solvent B and re-equilibrated for 5 min with 65% solvent B. An injection volume of 5 µL was used. A flow splitter directed the column effluent at approximately 0.2 mL/min into the mass spectrometer’s APCI (atmospheric pressure chemical ionization) ion source, operating in positive ion mode. The key parameters for the ion source included a capillary voltage of 4 kV, a corona current set to 6 µA, a nebulizer gas (N_2_) pressure of 2.5 bar, a drying gas (N_2_) flow rate of 3.0 L/min, a drying temperature of 250 °C, and an atmospheric pressure chemical ionization (APCI) heater temperature of 220 °C. Argon served as the collision gas, and the MS/MS collision energy was automatically adjusted between 2.5 and 35 eV, depending on the fragmented ion’s *m*/*z* value. Ion transfer parameters were optimized for the *m*/*z* range of 100 to 800, with a transfer time of 100 µs and pre-pulse storage of 10 µs. The data underwent automatic internal mass calibration using 5% (*w*/*v*) polyethylene glycol 4000 in 50% (*v*/*v*) 2-propanol, introduced into the ion source via a 20 µL loop at the beginning of each analysis.

After data acquisition and calibration, ion chromatograms for the protonated molecules of known ARs (Table 2) were extracted from the acquired scan data utilizing a width of 0.005 Da. Peaks of analytes were identified based on their molecular formulae (calculated from the [M+H]^+^ ions’ *m*/*z* with <5 ppm accuracy) and fragmentation spectra. Gaussian smoothing was applied to each chromatogram with a window width of 4 points and one iteration. Analyte peaks were integrated, and their areas were subsequently used for further calculations. Data acquisition and processing were done using Bruker DataAnalysis software, version 6.2, and Skyline, version 25.1.

Alkylresorcinol contents were estimated using a linear calibration curve (equation y = 8656.6x + 2346.2) created from a series of dilutions of the stock solution (1 mg/mL) of 5-n-heneicosylresorcinol (C21:0). The MS response demonstrated linearity within the concentration range of 2.5 to 200 ng/µL, with the limit of detection (LOD) calculated at 1.2 ng/µL and limit of quantification (LOQ) calculated at 3.6 ng/µL.

#### 2.8.3. Free Radical Scavenging Activity of ARs

The antiradical activity of alkylresorcinols in spring wheat cultivars was determined using a method previously devised and published for the first time in 2020 by Kowalska and Jędrejek [26]. Briefly, standard (*α*-tocopherol) and resorcinolic lipid fractions were transferred on a silica TLC plate. Plates were developed in vertical chambers using the mobile phase: toluene-ethyl acetate-formic acid (70:5:5, *v*/*v*/*v*). The plates were further processed as described in Section 2.7.4.

### 2.9. Statistical Analysis

The data were analyzed using PAST software v. 2022 [27]. In order to check the normality of the distributions, the Shapiro-Wilk test was used. In order to compare the influence of treatments (n = 4) and cultivars (n = 4) and their interaction effects on grain yield and health status, an analysis of variance ANOVA was made. Statistical analysis comparing plant protection variants’ influence on fungal pathogens’ infestation was done using the Kruskal-Wallis ANOVA test for nonparametric data sets. Dunn’s post-hoc test was used to identify the values of differences between variants. Mean values differing statistically at a significance level of *p* < 0.05 were marked with different lower-case letters of the alphabet. The results of PAs and ARs were subjected to statistical analysis using Statistica version 13 software (Stat. Soft. Inc., Tulsa, OK, USA) under multivariate analysis of variance and post-hoc analysis. Variation (ANOVA) was analyzed using the following experimental factors: year of cultivation, cultivars, and silicon treatments. The significance of differences was verified using Tukey’s test at *p* ≤ 0.05.

## 3. Results and Discussion

### 3.1. Effect of Silicon Treatments on the Yield and One Thousand Grain Weight

In both years of the study, a significant increase in yield compared to the control plant was obtained after a comprehensive seed dressing with AdeSil+ZumSil and two foliar treatments with ZumSil (variant B) (average of 8.9%) and two foliar treatments (average of 7.6%) (variant C) (Table 3). The least effective in terms of increasing yield was seed dressing with AdeSil+ZumSil (variant D).

In 2019, the applied silicon bio-preparations did not significantly differentiate thousand-grain weight (TGW) among the treatments, while in 2020, a significant increase in TGW compared to the control was found after a comprehensive seed dressing with AdeSil+ZumSil and two foliar treatments (variant B) (Table 4).

Significant differences in grain yield and TGW were found between the cultivars tested (Table 3 and Table 4). The spelt cv. Wirtas had the lowest yield, but also responded positively to the increased yield when using silicon treatments, especially in the comprehensive seed treatment technology in the variants with two foliar treatments (variant B). The cultivars of common wheat did not differ significantly in terms of yield.

The importance of silicon for the growth and yield of wheat is confirmed by other research [23,28,29]. Our research showed that the application of preparations with organic silicon increased the yield of the four spring wheat cultivars tested (cv. Harenda, Serenada, Rusałka, and spelt Wirtas), which is also confirmed by the results of Kowalska et al. [23]. The increase in yield compared to the control was the biggest when using comprehensive seed treatment technology in a variant with two foliar sprays and averaged 8.9% for the cultivars tested. Kowalska et al. [23] achieved an efficacy of 14 to 28% higher than the control for the cv. Rusałka and cv. Serenada using seed treatment and two foliar treatments.

In Huang et al. [30] sugarcane studies, combining soil and foliar Si application produced the best results. Guevel et al. [31] observed that the combined application was the most effective for wheat health. On the other hand, Segalin et al. [32] stated that the foliar application of silicon affected neither the yield, nor quality of the wheat grain of wheat cultivars.

### 3.2. Evaluation of Plant Infestation by Pathogens and Fusarium spp. Occurrence

Assessment of fungal disease infestation allowed the identification of differences between the resistance of wheat treated with different variants (A–D) of biological protection. Results were obtained from four cultivars assessed in the period of two years with completely different weather patterns (2019 and 2020). In 2019, the differences among the variants were particularly pronounced. The total infestation was the lowest in variant B for the cv. Harenda and cv. Rusałka, variant C for the cv. Wirtas and variant D for the cv. Serenada (Table 5). We can find as the most relevant finding, in most cases, that variants B, C, and D had lower fungal disease infestations than the control variant A. In 2019, brown rust on wheat leaves occurred in particularly high intensity. Plants grown in variant D proved to be the most resistant. However, the results varied depending on the cultivar. Septoria symptoms were not detected on the cv. Wirtas, except for treatment D. Other patterns of its occurrence seem to depend more on the wheat cultivar than on the treatment. The incidence of other symptoms (Tan spot, Mildew, Fusarium) was less pronounced and more dependent on the cultivar and the assessment year.

In 2020, the average flag leaf infestation (on the last evaluation date—10-th of July) did not exceed 5% (Table 5). In many cases, the control object had the best results. It can be concluded that in the vast majority of cases, different methods of plant protection did not improve the health of wheat in a year that can be considered optimal for its growth. In some cases, individual variants scored worse than the control, for example, variant D in the cv. Rusałka and cv. Serenada. Brown rust occurred in slightly higher intensity in variants B and D, while septoriasis was present mostly in variant C. Tan spot was most often found in variants D, A, and C. Powdery mildew and fusariosis practically did not occur in 2020 on the spring wheat tested (Table 5).

The results of the fungal diseases assessment in wheat do not indicate an increase in plant resistance due to the applied protective treatments. The selection of the appropriate cultivar for organic farming and the course of weather have a much greater impact on the health of the plantation [33]. However, in selected cases, it was possible to prove that different plant protection variants achieved a better result than the control object. This was the case in a situation of particularly high infection for more susceptible cultivars. The year 2019 was not particularly favorable for wheat health, as significant drought stress was observed, which increased the susceptibility of plants to other factors limiting the yield. Using any permitted form of plant protection brought a slight, but desirable effect in these conditions. Different situation was described in the year when the diseases occurred in low intensity. The protective measures applied did not bring any effect, often giving a result worse than the control. However, this does not allow draw conclusions that those treatments are unnecessary or harmful, as the total infestation was small compared to previous experiments in this location [34]. According to Artyszak [35], the foliar application of silicon had a stimulative effect on plants. However, the best results were observed in stressful conditions, such as salinity, a deficiency or excess of water, high and low temperatures, and the pressure of diseases and pests. In the study of Guevel et al. [31], foliar treatments with Si and nutrient salt solutions on wheat plants significantly reduced powdery mildew. In the study of Kowalska et al. [23], the severity of these diseases varied depending on the cultivar and the method of silicon application.

### 3.3. Quantitative and Qualitative Analysis of PAs and Their Antiradical Activity

**Figure 1:**
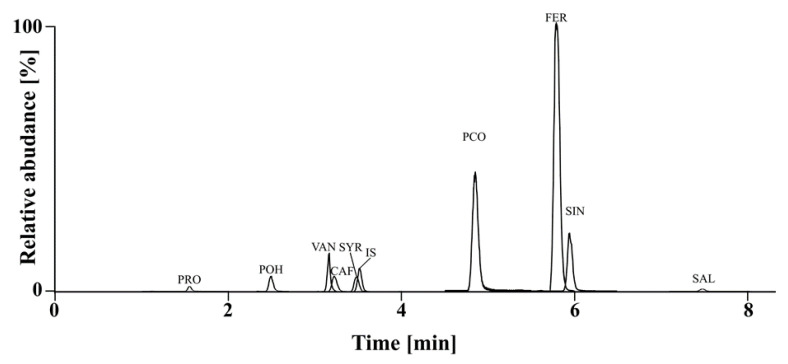
UPLC chromatograms in multiple reaction monitoring (MRM) mode of cv. Wirtas (2019) grain. Peak annotations: PRO—protocatechuic acid, POH—*p*-OH-benzoic acid, VAN—vanillic acid, CAF—caffeic acid, SYR—syringic acid, IS—Internal Standard (m-OH-benzoic acid), PCO—*p*-coumaric acid, FER—ferulic acid, SIN—sinapic acid, SAL—salicylic acid.

There are very little publications describing the effects of a cultivar and silicon treatments on phenolic acids content in organic spring wheat. The PAs content were analyzed using the UPLC-DAD-MS method. The nine phenolic acids: ferulic, protocatechuic, vanillic, *p*-OH-benzoic, caffeic, syringic, *p*-coumaric, salicylic, and sinapic acids were identified (Figure 1).

Ferulic and *p*-coumaric acids appeared to be the major phenolic acids in all samples. The other phenolic acids were present in much lower amounts, which can be represented as follows: FER > PCO > SIN > VAN > SYR > CAF > POH > PRO > SAL. Kowalska et al. [10] observed that the same three acids (FER, PCO and SIN) were also dominant in winter wheat as in this study. However, the content of the other phenolic acids was different in winter wheat (CAF > VAN > SYR > PRO > POH > SAL), while in the present study in spring wheat (VAN > SYR > CAF > POH > PRO > SAL). Ferulic acid represented for 58.7 to 79.2% of the total phenolic acids content of the wheat grains in 2019 and 57.5 to 78.9% of the total phenolic acids content of the wheat grains in 2020. Li et al. [36] showed a higher percentage content of ferulic acid (up to 84%) in the spring wheat cultivars. In our study, *p*-coumaric acid was 2.22–28.45% (in 2019) and 2.31–29.25% (in 2020) of total phenolic acids content.

The lowest amount was observed for salicylic acid, with a range of 0.16–0.21% (in 2019) and 0.16–0.20% (in 2020) of total phenolic acids content. The predominant PAs showed by Li et al. [36] in the spring wheat were ferulic, syringic, vanillic, and sinapic acids, whereas ferulic acid content for 130 wheat genotypes ranged from 326.0 to 1171.0 µg/g, with a mean concentration of 664.0 µg/g. Kurasiak-Popowska et al. [37] observed significant differences in ferulic acid content between 100 cultivars and lines of winter wheat. Its mean concentration was 975 µg/g, accounting for 92.4% of all phenolic acids.

Among the cultivars, the cv. Wirtas (*T. aestivum* L. ssp. *spelta* species) stood out. Except for sinapic acid, these cultivars contained significantly more other acids than other cultivars, e.g., *p*-coumaric acid contained more than 15 times more than other cultivars (Table S3). The cv. Wirtas contained a significantly higher total phenolic acid content (mean 1546.36 μg/g) than the cv. Rusałka, Serenada, and Harenda (mean 1018.02, 949.17, and 936.81 μg/g, respectively) (Table 6).

The highest content of the phenolic acids was observed in cv. Wirtas in other studies, too [9]. These cultivars also demonstrated the highest antiradical activity (Table 6). The year of the study did not affect the total phenolic acid content or the content of individual acids. The average total PAs content was 1111.05 μg/g (in 2019) and 1114.14 μg/g (in 2020). This is significantly more than in the research by Li et al. [36], where the mean total PAs content of spring wheat was 612 μg/g of dry matter (DM).

Seed treatment with AdeSil+ZumSil (variant D) increased the total phenolic acids content (1150.02 μg/g) compared to the other variants A, C, and B (1112.39, 1101.00 and 1086.96 μg/g, respectively). Except for sinapic acid, the use of variant D increased the content of the other acids and their activity compared to the other variants (Table 6). Silicon can act as a signal for the expression of genes that activate enzymes involved in the synthesis of polyphenols and flavonoids [38,39]. Fleck et al. [40] and Song et al. [41] showed that silicon uptake by plants under specific stress conditions, could increase the generation of phenolic compounds and antiradical defense enzymes. Kidd et al. [42] reported that silicon treatment enhanced the content of phenolic compounds in maize by 15 times compared to control plants. Dorneles et al. [43] study determined the effect of silicon on the accumulation of phenolic compounds, mainly ferulic, caffeic, syringic, and hydroxybenzoic acid in wheat. An increase in the concentration of these acids after silicon application has been demonstrated.

Phenolic compounds are important in plants, especially in the antiradical defense system [44]. Phenolic acids are natural antioxidants with possible health benefits [7]. Our results showed that many compounds with antiradical activity are present in the grain of the wheat cultivars studied, which may be of great health-promoting importance. Statistically significant differences (*p* < 0.05) in the antioxidant activity of individual cultivars were observed. The highest activity was shown by the cv. Wirtas (0.346, related to caffeic acid standard) and cv. Rusałka (0.227). In an earlier study by Kowalska et al. [6], the mean antiradical activity of winter wheat cultivars was lower (0.177). The antiradical activity of spring wheat grain results from ferulic, *p*-coumaric, sinapic, and vanillic acid content (Table 6). In the grains studied, ferulic acid was present in the highest amounts. It is the primary contributor to the total antioxidant activity because, after the DPPH^•^ scavenging process, a few semiquinones from this acid can be dimerized, increasing their activity. Chen et al. [7] showed that ferulic acid is more active than *p*-coumaric acid, although it contains only one methoxyl group. It can prevent diseases and promote good health through different physiological processes, as it is an excellent antioxidant. Moreover, Stumpf et al. [45] showed that ferulic acid and *p*-coumaric acid can even suppress the growth of fungal pathogens of wheat, such as *Fusarium* species. In our study, the highest antioxidant activity was shown after seed treatment with AdeSil+ZumSil (variant D). Higher antioxidant activity is an important factor, allowing the plant to tolerate abiotic stresses. Silicon activates the antiradical mechanisms to suppress the production of reactive oxygen species (ROS), and thus reduces lipid peroxidation [46]. A study by Aouz et al. [47] showed that silicon application significantly increased the levels of all antioxidants in wheat cultivars. Khan et al. [48] found that Si application increased antioxidant activity and neutralized oxidative stress in plants under high temperature and salinity conditions.

The results of the analysis of variance (ANOVA) are presented in Table 7.

The effect of year on the phenolic acids and their activity was not demonstrated. We observed that particular acid, total phenolic acid and activity were not sensitive to variation over the year. In contrast, the cultivar and silicon treatments significantly affected the studied parameters (Table 6). Year × cultivar interaction was significant for protocatechuic, ferulic, salicylic acid, total acids, and their activity. Total PAs content was the highest in cv. Wirtas, both in 2019 and 2020 (1542.33 and 1550.39 µg/g, respectively), followed by cv. Rusałka (1006.73 and 1029.31 µg/g) (Table S4). Year × silicon treatments interaction was significant only for salicylic acid. Cultivar × silicon treatments interaction was insignificant only for syringic acid (Table S5). The variability coefficient was low, ranging from 2.67% (for salicylic acid) to 6.24% (for *p*-OH-benzoic acid) (Table 7).

### 3.4. Quantitative and Qualitative Analysis of Alkylresorcinols (ARs, Resorcinolic Lipids) and Their Antiradical Activity

There is a lack of studies in the scientific literature describing the effect of the use of silicon treatments on the content of ARs in cereal grains. This is the first publication describing such a study.

**Figure 2:**
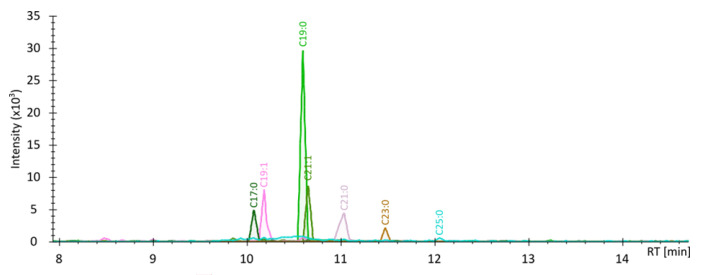
High-resolution extracted ion chromatograms (*m*/*z* of [M+H]^+^ ion ± 0.005) of seven ARs detected in the extract from the grain of cv. Wirtas (2019), variant A (control).

Seven AR derivatives: 5-*n*-heptadecylresorcinol (C17:0), 5-*n*-nonadecenylresorcinol (C19:1), 5-*n*-nonadecylresorcinol (C19:0), 5-*n*-heneicosenylresorcinol (C21:1), 5-*n*-heneicosylresorcinol (C21:0), 5-*n*-tricosylresorcinol (C23:0), and 5-*n*-pentacosylresorcinol (C25:0) were estimated using LC-APCI-MS (Figure 2).

As shown in Table S6, C21:0 and C19:0 homologues were present in the highest amount. C21:0 homologue represented 41.2–46.9% (in 2019) and 26.2–44.6% (in 2020), while C19:0 homologue was 24.2–31.2% (in 2019) and 27.8–40.2% (in 2020) of total ARs content. The lowest amount was observed for C25:0 homologue, with a range of 1.6–3.6% (in 2019) and 0.5–1.8% (in 2020). The concentration of ARs showed significant variability (*p* < 0.05) both between years, between cultivars, and according to the silicon treatments used. Mean ARs concentrations were higher in 2019 than in 2020 (566.49 and 351.18 μg/g, respectively) (Table 8). The total content of ARs in the different growth years ranged from 240.90 μg/g (cv. Wirtas, in 2020) to 692.40 μg/g (cv. Rusałka, in 2019) (Table S6). Among the cultivars, cv. Rusałka showed the highest mean total ARs content (570.31 μg/g of the grain), followed by cv. Harenda, Serenada, and Wirtas (524.73, 401.09, and 339.20 μg/g, respectively) (Table 8). The application of silicon treatments did not increase the content of ARs compared to the control. However, the best results, outside of the control, were obtained for variant C (469.29 μg/g), compared to variants D i B (449.81 and 439.27 μg/g, respectively).

Other studies on Polish spring wheat cultivars showed a statistically significant difference in ARs content (average 680 µg/g) [26]. In Polish winter wheat grains, the mean ARs concentration was about 723 μg/g [49] and 800 μg/g [50]. A much lower ARs content (239.8–234.0 mg/kg) in spring wheat was recorded by Zarnowski et al. [51]. These differences may result from different cultivars, environmental conditions, and analytical methods.

The results of the analysis of variance (ANOVA) are presented in Table 9.

Statistically significant differences were found in the content of individual ARs, their sum, and activity, depending on the year of study and cultivar. Silicon treatments had a significant effect on the studied ARs, except for C17:0 and C21:1. Years x cultivar interaction was not relevant only for C19:0 (Table S6). A varying effect of year on the level of ARs was monitored. It was statistically higher in 2019. Cultivars grown in 2019 also had significantly higher antioxidant activity (average 0.200 relative to α-tocopherol activity) than in 2020 (average 0.126). It can be supposed that the longer the alkyl chain, the greater the antioxidant activity related to AR. The results of the present research are in agreement with the result from the earlier publications by Kowalska and Jędrejek [26] and Kowalska et al. [9], which show that ARs possess poor antioxidant activity relative to tocopherols. Total ARs content was the highest in cv. Rusałka, both in 2019 and 2020 (average 570.32 µg/g), followed by cv. Harenda (average 524.74 µg/g) and cv. Serenade (average 401.09 µg/g). These cultivars also showed the highest antioxidant activity (0.201, 0.186, and 0.141, respectively). The high content of C19:0 and C21:0 derivatives may contribute to this, since the antioxidant activity is strongly influenced by factors, such as the length of the phenolic ring and the aliphatic side chain of the AR molecule [52]. The lowest total AR content was shown for the cv. Wirtas, in both years of the study (average 339.20 µg/g). This was in contrast to the phenolic acid content, where the cultivar had the highest content.

Year x silicon treatments interaction was significant for C17:0, C19:1, C19:0, C21:0, C23:0, total ARs and their activity. The highest content of individual ARs, their total amount, and activity were observed after two foliar treatments (in 2019) and in the control (variant A) in 2020 (Table S7). Cultivar × silicon treatments interaction was significant for C17:0, C19:1, C19:0, C21:0, C23:0, C25:0, total ARs and their activity. Cultivars responded differently to the silicon treatments used. The cv. Harenda showed the highest total ARs content and antioxidant activity in the control (variant A), the cv. Serenada and Rusałka—after two foliar treatments (variant C), while the cv. Wirtas—after seed dressing with AdeSil+ZumSil (variant D) (Table S8). The results showed that the antiradical activity of wheat grain showed a positive correlation with the total ARs content. The variability coefficient ranged from 6.92% (for C21:1) to 10.62% (for C23:0) (Table 9).

## 4. Conclusions

The research clearly indicates that the response of spring wheat to silicon treatments is strongly cultivar-dependent. The spelt cv. Wirtas, despite exhibiting the lowest baseline yield, showed a positive yield response to comprehensive silicon treatment and had the highest total PAs content. Conversely, the cv. Rusałka was characterized by the highest mean alkylresorcinol content. This highlights the necessity of selecting appropriate cultivars when implementing silicon-based technologies in organic farming systems.

This study is among the first to describe the effect of silicon treatments on ARs content in cereal grains. It was demonstrated that silicon treatments significantly modify the content of these compounds; however, the effect did not always mean an increase compared to the control and depended on multiple factors, including year, cultivar, and application variant interactions. This suggests a more complex mechanism of silicon’s influence on the metabolism of these phenolic lipids than in the case of phenolic acids.

The effectiveness of the tested silicon treatments, particularly regarding plant health and resistance to fungal diseases, was clearly linked to the weather conditions in a given year. The benefits of silicon application were more pronounced under stressful conditions (e.g., drought and high temperature in 2019), confirming silicon’s role in mitigating the effects of abiotic and biotic stresses. In both years of the study, a significant increase in the yield of spring wheat compared to the control was obtained after the application of a comprehensive seed stimulation technology with AdeSil+ZumSil preparations in variant with two foliar treatments with ZumSil (on average by 8.9%) and after only two foliar treatments (on average by 7.6%). Seed stimulation with AdeSil+ZumSil alone proved the least effective in increasing yield. Wheat responded with an increase in TGW after a comprehensive seed stimulation treatment with AdeSil+ZumSil and two foliar treatments in 2020. On average, variants B, C, and D provide better spring wheat resistance to fungal diseases in less favorable, dry, and hot years (2019). In optimal years for wheat growth, such as 2020, biological pest control treatments provide no additional benefit to spring wheat health. The control variant may perform as effectively or better in disease resilience. The tested silicon treatments, approved for use in organic farming, demonstrate potential not only for increasing yields, but also for modulating the content of important bioactive compounds in wheat grain. Further research could contribute to optimizing their application to maximize agronomic and health-promoting benefits in sustainable production systems.

## Figures and Tables

**Table 1:** Variants of bio-preparations with organic silicon (2019–2020).

Variant	Dosing of Treatments		Date of Application of Foliar Treatment
	Stimulation/Treatment of Seeds for Sowing	Foliar Treatments	
A—control	-	-	-
B—seed dressing with silicon treatments + foliar treatments with silicon preparations (2 ×)	AdeSil 0.5 kg per 100 kg of grain + 1% ZumSil solution to improve the stickiness of diatoms at a dose of 0.5 L per 100 kg of seeds	ZumSil 0.5 L/ha (2 ×)	BBCH 23–25 (3 to 5 tillers) BBCH 32–34 (third to fourth nodes)
C—only foliar treatments (2 ×)	-	ZumSil 0.5 L/ha (2 ×)	BBCH 23–25 (3 to 5 tillers) BBCH 32–34 (third to fourth nodes)
D—only seed dressing with silicon treatments AdeSil + ZumSil	AdeSil 0.5 kg per 100 kg of grain + 1% ZumSil solution to improve the stickiness of diatoms at a dose of 0.5 L per 100 kg of seeds	-	-

**Table 2:** Investigated alkylresorcinols (ARs).

Compound Name	Molecular Formula	Retention Time (min)	*m*/*z* of the [M+H]^+^ Ion
5-*n*-heptadecylresorcinol (C17:0)	C_23_H_40_O_2_	10.05	349.3101
5-*n*-nonadecenylresorcinol (C19:1)	C_25_H_42_O_2_	10.25	375.3258
5-*n*-nonadecylresorcinol (C19:0)	C_25_H_44_O_2_	10.53	377.3414
5-*n*-heneicosenylresorcinol (C21:1)	C_27_H_46_O_2_	10.67	403.3571
5-*n*-heneicosylresorcinol (C21:0)	C_27_H_48_O_2_	11.05	405.3727
5-*n*-tricosylresorcinol (C23:0)	C_29_H_52_O_2_	11.57	433.4040
5-*n*-pentacosylresorcinol (C25:0)	C_31_H_56_O_2_	12.06	461.4353

**Table 3:** Effect of silicon treatments on the yield (t/ha).

Cultivar	Silicon Treatments				Mean
	A	B	C	D	
2019				
Harenda	4.02 * ± 0.30 a (100.0%) ^#^	4.25 ± 0.25 a (105.7%)	4.21 ± 0.13 a (104.7%)	4.16 ± 0.41 a (103.5%)	4.16 ± 0.30 A
Serenada	3.84 ± 0.21 b (100.0%)	4.20 ± 0.40 ab (109.4%)	4.35 ± 0.10 a (113.2%)	4.15 ± 0.20 ab (108.1%)	4.14 ± 0.38 A
Rusałka	3.90 ± 0.28 b (100.0%)	4.36 ± 0.15 a (111.8%)	4.35 ± 0.31 a (115.4%)	4.20 ± 0.12 ab (107.7%)	4.20 ± 0.30 A
Wirtas	3.40 ± 0.29 c (100.0%)	3.70 ± 0.10 a (108.8%)	3.41 ± 0.35 bc (100.3%)	3.54 ± 0.13 a–c (104.1%)	3.51 ± 0.27 B
Mean	3.79 ± 0.40 b (100.0%)	4.13 ± 0.41 a (108.9%)	4.08 ± 0.50 a (107.7%)	4.01 ± 0.40 ab (105.8%)	4.00 ± 0.45
	2020				
Harenda	4.04 ± 0.51 b (100.0%)	4.79 ± 0.19 a (118.6%)	4.76 ± 0.35 a (117.8%)	4.37 ± 0.25 ab (108.2%)	4.49 ± 0.50 A
Serenada	3.95 ± 0.52 b (100.0%)	4.61 ± 0.30 a (116.7%)	4.49 ± 0.30 b (113.7%)	4.35 ± 0.17 b (110.1%)	4.35 ± 0.47 A
Rusałka	3.91 ± 0.15 b (100.0%)	4.49 ± 0.21 a (114.8%)	4.28 ± 0.10 b (109.5%)	4.33 ± 0.18 a (110.7%)	4.25 ± 0.30 A
Wirtas	3.13 ± 0.12 b (100.0%)	3.50 ± 0.14 a (111.8%)	3.47 ± 0.25 a (110.9%)	3.27 ± 0.10 b (104.5%)	3.34 ± 0.24 B
Mean	3.76 ± 0.60 c (100.0%)	4.34 ± 0.65 a (115.4%)	4.25 ± 0.71 b (113.1%)	4.08 ± 0.63 bc (108.5%)	4.10 ± 0.72

A—control untreated, B—seed dressing + 2 × foliar treatments, C—2 × foliar treatments, D—seed dressing. * Values followed by the same letter are not statistically different at *p* < 0.05. Values marked by different lowercase letters indicate differences between treatments, and values marked by capital letters indicate differences between cultivars. No significant interactions between treatments and cultivars were noted. ^#^ Values indicating the percentage increase are given in brackets.

**Table 4:** Effect of silicon treatments on the thousand-grain weight (TGW) (g).

Cultivar	Silicon Treatments				Mean
	A	B	C	D	
2019					
Harenda	35.5 * ± 0.3 a (100.0%) ^#^	35.6 ± 0.5 a (100.3%)	35.2 ± 0.9 a (99.2%)	35.4 ± 0.4 a (99.7%)	35.4 ± 0.5 C
Serenada	42.3 ± 0.7 a (100.0%)	42.1 ± 0.7 a (99.5%)	42.2 ± 0.7 a (99.8%)	42.7 ± 0.1 a (100.9%)	42.3 ± 0.6 B
Rusałka	41.3 ± 0.3 a (100.0%)	41.2 ± 0.7 a (99.8%)	41.6 ± 0.2 a (100.7%)	41.2 ± 0.7 a (99.8%)	41.3 ± 0.5 B
Wirtas **	58.1 ± 1.0 a (100.0%)	58.5 ± 0.9 a (100.7%)	59.0 ± 0.7 a (101.5%)	58.9 ± 0.8 a (101.4%)	58.6 ± 0.9 A
Mean	44.3 ± 8.6 a (100.0%)	44.3 ± 8.6 a (100.0%)	44.5 ± 1.2 a (100.5%)	44.5 ± 9.0 a (100.5%)	44.4 ± 8.7
	2020				
Harenda	36.9 ± 0.6 a (100.0%)	37.3 ± 0.6 a (101.1%)	36.7 ± 0.5 a (99.5%)	36.8 ± 0.2 a (99.7%)	36.9 ± 0.5 C
Serenada	42.6 ± 0.6 b (100.0%)	43.8 ± 0.8 a (102.8%)	43.7 ± 0.5 a (102.6%)	43.3 ± 0.6 ab (101.6%)	43.4 ± 0.7 B
Rusałka	38.5 ± 0.1 b (100.0%)	39.3 ± 0.5 a (102.1%)	38.9 ± 0.1 b (101.1%)	38.7 ± 0.5 b (100.5%)	38.9 ± 0.5 BC
Wirtas **	62.8 ± 0.2 b (100.0%)	64.4 ±1.5 a (102.5%)	63.2 ± 0.3 b (100.6%)	63.2 ± 0.3 b (100.6%)	63.4 ± 0.9 A
Mean	45.2 ± 10.7 b (100.0%)	46.2 ± 11.1 a (102.2%)	45.6 ± 10.8 b (100.9%)	45.5 ± 10.9 b (100.7%)	45.6 ± 10.6

A—control untreated, B—seed dressing + 2 × foliar treatment, C—2 × foliar treatments, D—seed dressing. * Values followed by the same letter are not statistically different at *p* < 0.05. Values marked by different lowercase letters indicate differences between treatments, and values marked by capital letters indicate differences between cultivars. No significant interactions between treatments and cultivars were noted. ^#^ Values indicating the percentage increase are given in brackets. ** weight of hulled grain.

**Table 5:** Wheat leaf infestation (%) by various fungal diseases in 2019 and 2020.

		Total Infestation	Brown Rust	Septoria Blotch	Tan Wpot	Mildew	Fusarium Wilt
2019							
Harenda	A	2.50 ± 1.24 ab	1.00 ± 0.00 b	0.80 ± 0.89 b	0.70 ± 0.80 a	0.00 ± 0.00 b	0.00 ± 0.00 b
	B	2.00 ± 1.17 b	1.25 ± 0.55 ab	0.40 ± 0.75 bc	0.00 ± 0.00 b	0.00 ± 0.00 b	0.35 ± 0.67 a
	C	2.55 ± 0.89 ab	1.50 ± 0.69 a	0.00 ± 0.00 c	0.70 ± 0.80 a	0.00 ± 0.00 b	0.35 ± 0.67 a
	D	3.85 ± 2.50 a	1.00 ± 0.00 b	1.75 ± 1.62 a	0.35 ± 0.67 ab	0.50 ± 0.89 a	0.25 ± 0.64 ab
Serenada	A	15.20 ± 11.7 ab	12.50 ± 12.5 ab	0.80 ± 0.89 a	0.35 ± 0.67 a	0.50 ± 0.89 a	1.05 ± 0.76 ab
	B	16.65 ± 20.8 a	14.00 ± 21.64 a	0.40 ± 0.75 ab	0.35 ± 0.67 a	0.50 ± 0.89 a	1.40 ± 0.50 a
	C	9.10 ± 8.78 b	7.50 ± 7.65 ab	0.40 ± 0.75 ab	0.00 ± 0.00 a	0.50 ± 0.89 a	0.70 ± 0.80 b
	D	2.35 ± 0.99 c	2.00 ± 0.65 b	0.00 ± 0.00 b	0.35 ± 0.67 a	0.00 ± 0.00 b	0.00 ± 0.00 c
Rusałka	A	59.30 ± 15.75 a	57.50 ± 16.42 a	1.75 ± 1.62 a	0.05 ± 0.22 b	0.00 ± 0.00 a	0.00 ± 0.00 c
	B	41.75 ± 10.62 b	41.00 ± 11.19 b	0.40 ± 0.75 b	0.00 ± 0.00 b	0.00 ± 0.00 a	0.35 ± 0.67 b
	C	54.45 ± 20.09 a	53.00 ± 19.96 a	0.40 ± 0.75 b	0.70 ± 0.80 a	0.00 ± 0.00 a	0.35 ± 0.67 b
	D	60.50 ± 16.77 a	57.50 ± 16.42 a	1.60 ± 0.50 a	0.00 ± 0.00 b	0.00 ± 0.00 a	1.40 ± 0.50 a
Wirtas	A	29.35 ± 7.44 a	29.00 ± 7.18 a	0.00 ± 0.00 b	0.35 ± 0.67 b	0.00 ± 0.00 a	0.00 ± 0.00 a
	B	23.35 ± 6.47 b	23.00 ± 6.57 b	0.00 ± 0.00 b	0.35 ± 0.67 b	0.00 ± 0.00 a	0.00 ± 0.00 a
	C	14.05 ± 9.44 c	13.00 ± 8.80 c	0.00 ± 0.00 b	1.05 ± 0.76 a	0.00 ± 0.00 a	0.00 ± 0.00 a
	D	21.40 ± 8.84 bc	21.00 ± 8.21 b	0.40 ± 0.75 a	0.00 ± 0.00 c	0.00 ± 0.00 a	0.00 ± 0.00 a
	2020						
Harenda	A	1.72 ± 2.04 b	0.05 ± 0.21 a	1.35 ± 2.06 a	0.05 ± 0.30 a	0.28 ± 0.70 a	0.00 ± 0.00 a
	B	2.83 ± 3.46 a	0.27 ± 1.57 a	2.02 ± 3.09 a	0.10 ± 0.44 a	0.44 ± 0.84 a	0.00 ± 0.00 a
	C	2.87 ± 2.85 a	0.03 ± 0.16 a	2.08 ± 2.61 a	0.15 ± 0.54 a	0.44 ± 1.05 a	0.18 ± 0.85 a
	D	2.59 ± 2.27 ab	0.00 ± 0.00 a	1.80 ± 1.96 a	0.44 ± 1.64 a	0.29 ± 0.72 a	0.05 ± 0.31 a
Serenada	A	3.02 ± 1.84 a	0.10 ± 0.49 a	0.73 ± 1.30 a	0.20 ± 0.60 b	2.00 ± 1.94 a	0.00 ± 0.00 a
	B	2.00 ± 1.59 b	0.08 ± 0.27 a	0.10 ± 0.44 b	0.18 ± 0.84 b	1.65 ± 1.46 a	0.00 ± 0.00 a
	C	2.88 ± 1.58 a	0.24 ± 0.69 a	0.52 ± 0.89 a	0.38 ± 0.79 ab	1.74 ± 1.52 a	0.00 ± 0.00 a
	D	3.73 ± 3.32 a	0.12 ± 0.33 a	1.15 ± 2.72 a	0.63 ± 1.28 a	1.83 ± 1.72 a	0.00 ± 0.00 a
Rusałka	A	2.75 ± 2.47 b	0.35 ± 1.59 b	1.80 ± 2.17 b	0.00 ± 0.00 a	0.60 ± 1.28 a	0.00 ± 0.00 a
	B	4.05 ± 4.23 ab	0.63 ± 1.03 a	3.18 ± 3.88 ab	0.00 ± 0.00 a	0.25 ± 0.67 a	0.00 ± 0.00 a
	C	5.03 ± 4.91 a	0.45 ± 0.75 ab	4.33 ± 4.76 a	0.05 ± 0.32 a	0.20 ± 0.61 a	0.00 ± 0.00 a
	D	4.95 ± 4.81 a	1.44 ± 1.93 a	2.92 ± 4.34 ab	0.00 ± 0.00 a	0.59 ± 1.12 a	0.00 ± 0.00 a
Wirtas	A	1.66 ± 2.33 ab	0.68 ± 0.91 c	0.27 ± 0.92 b	0.37 ± 0.99 a	0.34 ± 1.61 a	0.00 ± 0.00 a
	B	2.58 ± 2.85 a	1.58 ± 1.87 a	0.78 ± 1.85 a	0.05 ± 0.32 b	0.18 ± 0.84 a	0.00 ± 0.00 a
	C	1.55 ± 1.34 b	0.78 ± 0.80 bc	0.60 ± 0.93 ab	0.05 ± 0.32 b	0.13 ± 0.79 a	0.00 ± 0.00 a
	D	2.59 ± 2.56 a	1.41 ± 1.45 ab	0.80 ± 2.03 a	0.20 ± 0.60 ab	0.17 ± 0.83 a	0.00 ± 0.00 a

A—control untreated, B—seed dressing + 2 × foliar treatments, C—2 × foliar treatments, D—seed dressing. Values followed by the same letter are not statistically different at *p* < 0.05. Values marked by different lowercase letters indicate differences between treatments within a cultivar.

**Table 6:** Influence of the year, cultivar, and silicon treatments on phenolic acid content and antioxidant activity. Mean phenolic acids (µg/g of the grain), total phenolic acids concentration (µg/g of the grain), and antiradical activity (in relation to caffeic acid’s activity = 1.00) of four spring wheat cultivars.

Phenolic Acid	Year		Cultivar				Silicon Treatments			
	2019	2020	Harenda	Serenada	Rusałka	Wirtas	A	B	C	D
Protocatechuic acid	3.75 ± 0.17 a	3.73 ± 0.16 a	3.18 ± 0.03 c	3.31 ± 0.02 b	3.30 ± 0.03 bc	5.18 ± 0.31 a	3.27 ± 0.03 d	3.80 ± 0.22 b	3.57 ± 0.11 c	4.33 ± 0.36 a
*p*-OH-Benzoic acid	8.54 ± 0.39 a	8.53 ± 0.40 a	5.58 ± 0.05 d	8.91 ± 0.08 b	7.05 ± 0.09 c	12.60 ± 0.21 a	8.63 ± 0.57 ab	8.28 ± 0.47 b	8.52 ± 0.56 ab	8.71 ± 0.63 a
Vanillic acid	34.03 ±1.41 a	34.55 ±1.43 a	26.03 ± 0.27 d	29.04 ± 0.18 c	31.53 ± 0.25 b	50.56 ± 0.59 a	33.92 ± 1.99 b	33.83 ± 1.87 b	33.80 ± 2.08 b	35.61 ± 2.16 a
Caffeic acid	29.19 ± 0.32 a	29.62 ± 0.32 a	26.79 ± 0.40 c	30.87 ± 0.29 a	30.19 ± 0.34 ab	29.76 ± 0.26 b	29.67 ± 0.27 ab	28.13 ± 0.56 c	29.37 ± 0.40 b	30.44 ± 0.43 a
Syringic acid	29.56 ± 1.06 a	29.60 ± 1.06 a	21.51 ± 0.21 d	26.97 ± 0.25 c	28.87 ± 0.25 b	40.98 ± 0.44 a	28.69 ± 1.45 c	29.16 ± 1.44 bc	29.73 ± 1.47 b	30.75 ± 1.66 a
*p*-Coumaric acid	123.81 ± 25.13 a	124.75 ± 25.34 a	22.22 ± 0.28 c	23.21 ± 0.29 bc	28.78 ± 0.39 b	422.91 ± 5.96 a	121.84 ± 35.45 b	120.93 ± 34.90 b	118.66 ± 33.86 b	135.70 ± 39.73 a
Ferulic acid	795.60 ± 12.67 a	795.82 ± 12.73 a	729.94 ± 9.02 c	736.18 ± 7.45 c	795.44 ± 6.07 b	921.27 ± 10.82 a	798.19 ± 24.16 b	776.42 ± 12.81 c	789.51 ± 13.59 bc	818.72 ± 18.58 a
Sinapic acid	84.56 ± 2.26 a	85.51 ± 2.20 a	99.82 ± 0.54 a	89.02 ± 0.43 b	91.10 ± 0.91 b	60.20 ± 0.91 c	86.19 ± 2.85 a	84.41 ± 3.56 ab	85.83 ± 2.93 ab	83.70 ± 3.34 b
Salicylic acid	2.02 ± 0.07 a	2.02 ± 0.07 a	1.76 ± 0.03 b	1.66 ± 0.00 c	1.77 ± 0.02 b	2.90 ± 0.04 a	1.99 ± 0.10 b	1.99 ± 0.10 b	2.01 ± 0.10 b	2.09 ± 0.13 a
Total	1111.05 ± 37.47 a	1114.14 ± 37.67 a	936.81 ± 9.51 c	949.17 ± 8.10 c	1018.02 ± 7.44 b	1546.36 ± 15.91 a	1112.39 ± 59.51 b	1086.96 ± 47.16 c	1101.00 ± 47.50 bc	1150.02 ± 58.50 a
Antiradical activity	0.248 ± 0.008 a	0.249 ± 0.008 a	0.209 ± 0.002 c	0.212 ± 0.002 c	0.227 ± 0.002 b	0.346 ± 0.004 a	0.250 ± 0.013 b	0.243 ± 0.011 c	0.244 ± 0.011 c	0.257 ± 0.013 a

Values belonging to the same traits without common lowercase letters are statistically different in rows for each year, cultivar, and silicon treatments.

**Table 7:** Summary of analysis of variance results. Significance of effect (years, cultivars, and silicon treatments) in the analysis of variance and variability coefficient of phenolic acids.

Phenolic Acid	Source of Variability							V (%) ^#^
	Year (Y)	Cultivar (C)	Silicon Treatments (S)	Y × C	Y × S	C × S	Y × C × S	
Protocatechuic acid	ns	***	***	**	ns	***	ns	4.47
*p*-OH-Benzoic acid	ns	***	*	ns	ns	***	ns	6.24
Vanillic acid	ns	***	***	ns	ns	*	ns	4.52
Caffeic acid	ns	***	***	ns	ns	***	ns	4.23
Syringic acid	ns	***	***	ns	ns	ns	ns	4.19
*p*-Coumaric acid	ns	***	***	ns	ns	***	ns	6.02
Ferulic acid	ns	***	***	***	ns	***	***	3.03
Sinapic acid	ns	***	*	ns	ns	***	ns	3.47
Salicylic acid	ns	***	***	***	***	***	***	2.67
Total	ns	***	***	***	ns	***	ns	2.92
Antiradical activity	ns	***	***	***	ns	***	ns	3.22

Significance levels are *** *p* < 0.001, ** *p* < 0.01; * *p* < 0.05, ns—not significant; ^#^ Variability coefficient V (%) = √S^2^/x × 100%.

**Table 8:** Influence of the year, cultivar, and silicon treatments on AR content and antioxidant activity. Mean ARs (µg/g of the grain), total phenolic acids concentration (µg/g of the grain), and antiradical activity (in relation to α-tocopherol’s activity = 1.00) of four spring wheat cultivars.

Alkyl- Resorcinol	Year		Cultivar				Silicon Treatments			
	2019	2020	Harenda	Serenada	Rusałka	Wirtas	A	B	C	D
C17:0	22.66 ± 0.43 a	17.21 ± 0.75 b	21.78 ± 0.70 b	18.62 ± 0.80 c	24.18 ± 0.65 a	15.16 ± 0.90 d	20.55 ± 1.03 a	19.37 ± 0.97 a	20.34 ± 1.13 a	19.49 ± 1.01 a
C19:1	37.45 ±1.11 a	15.92 ± 0.59 b	29.03 ± 2.37 b	24.42 ± 1.99 c	33.07 ± 3.04 a	20.22 ± 1.94 d	26.46 ± 2.35 ab	25.58 ± 2.43 b	27.81 ± 2.90 a	26.89 ± 2.58 ab
C19:0	160.30 ± 4.93 a	121.41 ± 4.99 b	159.76 ± 5.03 b	128.30 ± 5.65 c	177.35 ± 5.75 a	98.01 ± 4.04 d	145.21 ± 8.31 ab	135.09 ± 7.02 b	145.58 ± 9.09 a	137.54 ± 7.88 ab
C21:1	31.24 ± 0.44 b	49.83 ± 1.54 a	38.95 ± 1.97 b	36.67 ± 1.77 b	45.84 ± 3.11 a	40.69 ± 2.61 ab	41.55 ± 2.93 a	38.27 ± 2.32 a	40.08 ± 1.82 a	42.24 ± 2.79 a
C21:0	250.73 ± 7.92 a	125.64 ± 7.33 b	224.99 ± 12.37 b	157.15 ± 14.15 c	239.77 ± 17.04 a	130.83 ± 12.37 d	197.60 ± 16.57 a	179.94 ± 15.37 b	192.74 ± 19.30 ab	182.46 ± 16.25 b
C23:0	51.30 ± 1.29 a	17.41 ± 1.26 b	40.50 ± 3.49 b	29.21 ± 3.67 c	41.89 ± 4.17 a	25.83 ± 3.43 d	36.74 ± 3.86 a	32.99 ± 3.69 b	34.51 ± 4.44 ab	33.19 ± 3.81 b
C25:0	12.81 ± 0.23 a	3.75 ± 0.28 b	9.72 ± 0.86 a	6.72 ± 0.94 c	8.22 ± 0.91 b	8.45 ± 1.20 b	8.86 ± 1.05 a	8.03 ± 0.97 b	8.22 ± 1.02 b	8.01 ± 0.98 b
Total	566.49 ± 15.79 a	351.18 ± 15.17 b	524.73 ± 23.09 b	401.09 ± 25.94 c	570.31 ± 28.51 a	339.20 ± 21.46 d	476.96 ± 30.65 a	439.27 ± 28.80 b	469.29 ± 36.00 a	449.81 ± 29.52 ab
Antiradical activity	0.200 ± 0.006 a	0.126 ± 0.005 b	0.186 ± 0.008 b	0.141 ± 0.009 c	0.201 ± 0.010 a	0.124 ± 0.007 d	0.168 ± 0.011 a	0.155 ± 0.010 b	0.166 ± 0.013 a	0.162 ± 0.009 ab

Values belonging to the same traits without common lowercase letters are statistically different in rows for each year, cultivar, or silicon treatments.

**Table 9:** Summary of analysis of variance results. Significance of effect (years, cultivars, and silicon treatments) in the analysis of variance and variability coefficient of alkylresorcinols.

Alkyl- Resorcinol	Source of Variability							V (%) ^#^
	Year (Y)	Cultivar (C)	Silicon Treatments (S)	Y × C	Y Y	C × S	Y × C × S	
C17:0	***	***	ns	***	*	*	ns	10.33
C19:1	***	***	*	***	*	*	*	10.23
C19:0	***	***	*	ns	*	*	ns	9.50
C21:1	***	***	ns	*	ns	ns	ns	6.92
C21:0	***	***	**	***	***	***	***	8.95
C23:0	***	***	**	**	**	***	***	10.62
C25:0	***	***	***	***	ns	***	***	8.83
Total	***	***	**	*	**	***	**	8.28
Antiradical activity	***	***	**	**	**	***	***	7.98

Significance levels are *** *p* < 0.001, ** *p* < 0.01; * *p* < 0.05, ns—insignificant. ^#^ Variability coefficient V (%) = √S2/x × 100%.

## Data Availability

The original contributions presented in this study are included in the article. Further inquiries can be directed to the corresponding author(s).

## Supplementary Materials

The following supporting information can be downloaded at: https://www.mdpi.com/article/10.3390/foods14142406/s1, Table S1. Meteorological conditions in Grabów in the growing seasons of 2019–2020; Table S2. Calibration curve parameters for nine phenolic acids; Table S3. Mean phenolic acids (µg/g of the grain ± SD), total phenolic acids content (µg/g of the grain ± SD) and antiradical activity (in relation to caffeic acid’s activity = 1.00) of spring wheat cultivars; Table S4. Influence of cultivar for phenolic acid content (µg/g of the grain) and antiradical activity (in relation to caffeic acid’s activity = 1.00), in the study years (2019–2020); Table S5. Influence of cultivar for phenolic acid content (µg/g of the grain) and antiradical activity (in relation to caffeic acid’s activity = 1.00) dependent on silicon treatments; Table S6. Mean alkylresorcinols (µg/g of the grain ± SD), total alkylresorcinols content (µg/g of the grain ± SD) and antiradical activity (in relation to *α*-tocopherol’s activity = 1.00) of four spring wheat cultivars; Table S7. Influence of silicon treatments (S) for alkylresorcinols content (µg/g of the grain) and antioxidant activity (in relation to α-tocopherol’s activity = 1.00), in the study years (2019–2020); Table S8. Influence of cultivars (C) and silicon treatments (S) for alkylresorcinol content (µg/g of the grain) and antioxidant activity (in relation to α-tocopherol’s activity = 1.00) of spring wheat cultivars; Figure S1. Field experiment on the effect of silica biopreparations on healthiness, yield and quality of spring wheat grain in Grabów (Poland).

## Abbreviations

Photodiode array detector (PDA), liquid chromatography (LC), mass spectrometry (MS), quadrupole mass detector (TQD), multiple reaction monitoring (MRM), quadrupole-time of flight (Q-TOF), atmospheric pressure chemical ionization (APCI), alkylresorcinols (ARs), 5-*n*-heptadecylresorcinol (C17:0), 5-*n*-nonadecenylresorcinol (C19:1), 5-*n*-nonadecylresorcinol (C19:0), 5-n-heneicosenylresorcinol (C21:1), 5-*n*-heneicosylresorcinol (C21:0), 5-*n*-tricosylresorcinol (C23:0), 5-*n*-pentacosylresorcinol (C25:0), sodium hydroxide (NaOH), hydrochloric acid (HCl), thin-layer chromatography (TLC), 2,2-diphenyl-1-picrylhydrazyl radical (DPPH^•^), ultra-performance liquid chromatography (UPLC), phenolic acids (PAs), protocatechuic acid (PRO), *p*-OH-benzoic acid (POH), vanillic acid (VAN), caffeic acid (CAF), syringic acid (SYR), internal standard (IS), *p*-coumaric acid (PCO), ferulic acid (FER), sinapic acid (SIN), salicylic acid (SAL), Institute of Soil Science and Plant Cultivation—State Research Institute (IUNG-PIB), Biologische Bundesanstalt, Bundessortenamt und CHemische Industrie scale (BBCH scale), analysis of variance (ANOVA), limit of quantification (LOQ), limit of detection (LOD), dry matter (DM), cultivar (cv.), standard deviation (SD), thousand grain yield (TGW), reactive oxygen species (ROS).
