# Supplementary material for: The Effects of a Cultivar and Silicon Treatments on Grain Parameters and Bioactive Compound Content in Organic Spring Wheat
Source: Foods. 2025 Jul 8;14(14):2406. doi: 10.3390/foods14142406 (PMC12294375; doi:10.3390/foods14142406)
Supplement: Supplementary file 1 [file foods-14-02406-s001.zip › foods-3697944-supplementary.pdf]

SUPPLEMENTARY MATERIALS

The Effects of a Cultivar and Silicon Treatments on Grain Parameters and Bioactive Compound Content in Organic Spring Wheat

Iwona Kowalska [1,*], Mariusz Kowalczyk [1], Jarosław Mołdoch [1], Sylwia Pawelec [1], Paweł Radzikowski [2] and Beata Feledyn-Szewczyk [2]

[1] Department of Phytochemistry, Institute of Soil Science and Plant Cultivation-State Research Institute, Czartoryskich Str. 8, 24-100 Pulawy, Poland; ikowalska@iung.pulawy.pl (I.K.); jmoldoch@iung.pulawy.pl (J.M.); spawelec@iung.pulawy.pl (S.P.)

[2] Department of Agroecology and Economics, Institute of Soil Science and Plant Cultivation-State Re-search Institute, Czartoryskich Str. 8, 24-100 Pulawy, Poland; pradzikowski@iung.pulawy.pl (P.R.), bszewczyk@iung.pulawy.pl (B.F.-S.)

* Correspondence: ikowalska@iung.pulawy.pl; Tel.: +48-81-4786883

Table and Figure of Contents:

Table S1. Meteorological conditions in Grabów in the growing seasons of 2019-2020.

Table S2. Calibration curve parameters for nine phenolic acids.

Table S3. Mean phenolic acids (µg/g of the grain ± SD), total phenolic acids content (µg/g of the grain ± SD) and antiradical activity (in relation to caffeic acid's activity = 1.00) of spring wheat cultivars.

Table S4. Influence of cultivar for phenolic acid content (µg/g of the grain) and antiradical activity (in relation to caffeic acid's activity = 1.00), in the study years (2019-2020).

Table S5. Influence of cultivar for phenolic acid content (µg/g of the grain) and antiradical activity (in relation to caffeic acid's activity = 1.00) dependent on silicon treatments.

Table S6. Mean alkylresorcinols (µg/g of the grain ± SD), total alkylresorcinols content (µg/g of the grain ± SD) and antiradical activity (in relation to α-tocopherol's activity = 1.00) of four spring wheat cultivars.

Table S7. Influence of silicon treatments (S) for alkylresorcinols content (µg/g of the grain) and antioxidant activity (in relation to *α*-tocopherol's activity = 1.00), in the study years (2019-2020).

Table S8. Influence of cultivars (C) and silicon treatments (S) for alkylresorcinol content (µg/g of the grain) and antioxidant activity (in relation to *α*-tocopherol's activity = 1.00) of spring wheat cultivars.

Figure S1. Field experiment on the effect of silica biopreparations on healthiness, yield and quality of spring wheat grain in Grabów (Poland).

Table of Contents:

Table S1. Meteorological conditions in Grabów in the growing seasons of 2019-2020.

**Month**	**Mean Temperaturę (°C)**			**Rainfall (mm)**		
	**2019**	**2020**	**Multi-Annual Average**	**2019**	**2020**	**Multi-Annual Average**
March	5.4	4.5	5.0	22.2	20.0	25.0
April	9.8	8.6	7.5	37.5	15.6	42.0
May	13.1	11.3	12.4	51.5	76.5	53.0
June	21.7	18.3	16.7	51.2	157.8	110.0
July	18.7	18.6	17.8	20.2	38.3	105.0

Table S2. Calibration curve parameters for nine phenolic acids.

No.	Phenolic Acid	Calibration Curve	R^2
1	protocatechuic acid (PRO)	$y = -0.0254x^2 + 1.4661x + 0.0138$	0.992
2	*p*-hydroxybenzoic acid (POH)	$y = -0.0117x^2 + 1.4390x + 0.1650$	0.998
3	vanillic acid (VAN)	$y = 0.0001x^2 + 0.1940x - 0.0031$	0.999
4	caffeic acid (CAF)	$y = -0.0183x^2 + 2.4211x + 0.4368$	0.997
5	syryngic acid (SYR)	$y = -0.0001x^2 + 0.2598x - 0.0026$	0.999
6	*p*-coumaric acid (PCO)	$y = -0.0166x^2 + 2.0582x + 2.0582$	0.980
7	ferulic acid (FER)	$y = -0.0004x^2 +0.3801x + 3.3001$	0.980
8	sinapinic acid (SIN)	$y = -0.0032x^2 +0.5524x - 0.0620$	0.996
9	salicylic acid (SAL)	$y = -0.0338x^2 +3.2638x + 0.8268$	0.996

Table S3. Mean phenolic acids (µg/g of the grain ± SD), total phenolic acids content (µg/g of the grain ± SD) and antiradical activity (in relation to caffeic acid's activity = 1.00) of spring wheat cultivars.

Cultivar	Year	Protocate-chuic Acid	*p*-OH-Benzoic Acid	Vanillic Acid	Caffeic Acid	Syringic Acid	*p*-Coumaric Acid	Ferulic Acid	Sinapic Acid	Salicylic Acid	Total	Antiradical Activity
Harenda	2019	3.25 ± 0.04bc	5.65 ± 0.07d	25.78 ± 0.36d	26.80 ± 0.58b	21.51 ± 0.30e	22.83 ± 0.46b	759.22 ± 11.29c	99.24 ± 0.91a	1.88 ± 0.01b	966.17 ± 12.78cd	0.216 ± 0.00d
	2020	3.11 ± 0.01c	5.51 ± 0.07d	26.27 ± 0.41d	26.77 ± 0.57b	21.51 ± 0.30e	21.60 ± 0.22b	700.67 ± 7.56d	100.39 ± 10.59a	1.63 ± 0.01c	907.46 ± 7.65e	0.202 ± 0.00e
Serenada	2019	3.37 ± 0.02b	8.90 ± 0.10b	28.92 ± 0.24c	30.82 ± 0.45a	27.25 ± 0.44cd	22.69 ± 0.48b	716.29 ± 11.88d	89.07 ± 0.57b	1.65 ± 0.01c	928.95 ± 13.10de	0.208 ± 0.00d
	2020	3.25 ± 0.01bc	8.91 ± 0.12b	29.17 ± 0.27c	30.93 ± 0.39a	26.69 ± 0.26d	23.73 ± 0.26b	756.07 ± 4.33c	88.97 ± 0.66b	1.67 ± 0.00c	969.40 ± 5.31cd	0.217 ± 0.00cd
Rusałka	2019	3.20	7.01	31.37	29.69	28.59	28.69	785.92	90.62	1.65	1006.73	0.223

		± 0.03bc	± 0.14c	± 0.34b	± 0.52a	± 0.40bc	± 0.60b	± 10.38bc	± 1.32b	± 0.01c	± 12.92bc	± 0.00bc
	2020	3.39 ± 0.01b	7.09 ± 0.12c	31.68 ± 0.38b	30.69 ± 0.40a	29.15 ± 0.30b	28.88 ± 0.51b	804.95 ± 5.47b	91.58 ± 1.29b	1.88 ± 0.01b	1029.31 ± 6.45b	0.231 ± 0.00b
Wirtas	2019	5.17 ± 0.47a	12.59 ± 0.29a	50.04 ± 1.02a	29.44 ± 0.35a	40.91 ± 0.65a	421.02 ± 9.29a	920.96 ± 15.45a	59.30 ± 1.24c	2.90 ± 0.06a	1542.33 ± 25.87a	0.345 ± 0.01a
	2020	5.19 ± 0.44a	12.60 ± 0.31a	51.09 ± 0.59a	30.07 ± 0.38a	41.06 ± 0.61a	424.80 ± 7.85a	921.58 ± 15.86a	61.09 ± 1.34c	2.90 ± 0.06a	1550.39 ± 19.64a	0.347 ± 0.00a

Values are expressed as mean ± SD. Means in a column followed by different letters show significant differences (p < 0.05) according to the Tukey test.

Table S4. Influence of cultivar for phenolic acid content (µg/g of the grain) and antiradical activity (in relation to caffeic acid's activity = 1.00), in the study years (2019-2020).

**Phenolic Acid**	**2019**				**2020**			
	**Harenda**	**Serenada**	**Rusałka**	**Wirtas**	**Harenda**	**Serenada**	**Rusałka**	**Wirtas**
PRO	3.25 ± 0.04b	3.37 ± 0.02b	3.20 ± 0.03b	5.17 ± 0.47a	3.11 ± 0.01b	3.25 ± 0.01b	3.39 ± 0.01b	5.19 ± 0.44a
FER	759.22 ± 11.29b	716.29 ± 11.88c	785.92 ± 10.38b	920.96 ± 15.45a	700.67 ± 7.56d	756.07 ± 4.33c	804.95 ± 5.47b	921.58 ± 15.86a
SAL	1.88 ± 0.01b	1.65 ± 0.01c	1.65 ± 0.01c	2.90 ± 0.05a	1.63 ± 0.01c	1.67 ± 0.00c	1.88 ± 0.01b	2.90 ± 0.06a
Total	966.17 ± 12.78c	928.95 ± 13.10d	1006.73 ± 12.92b	1542.33 ± 25.87a	907.46 ± 7.65d	969.40 ± 5.31c	1029.31 ± 6.45b	1550.39 ± 19.64a
Antiradical activity	0.216 ± 0.00c	0.208 ± 0.00c	0.223 ± 0.00b	0.345 ± 0.01a	0.202 ± 0.00d	0.217 ± 0.00c	0.231 ± 0.00b	0.347 ± 0.01a

Comparison of averages for combinations of years (Y) * cultivars (C) – C/Y. Values are expressed as mean ± SD. Means in a row followed by different letters show significant differences (p < 0.05) according to the Tukey test. PRO - protocatechuic acid, FER - ferulic acid, SAL - salicylic acid.

Table S5. Influence of cultivar for phenolic acid content (µg/g of the grain) and antiradical activity (in relation to caffeic acid's activity = 1.00) dependent on silicon treatments.

Phenolic acid	Harenda				Serenada			
	A	B	C	D	A	B	C	D
PRO	3.23 ± 0.08a	3.08 ± 0.01b	3.17 ± 0.02a	3.25 ± 0.04a	3.31 ± 0.05a	3.27 ± 0.03a	3.31 ± 0.04a	3.35 ± 0.03a
POH	5.76 ± 0.02a	5.54 ± 0.06a	5.36 ± 0.06b	5.66 ± 0.16a	8.83 ± 0.16b	9.07 ± 0.12a	8.88 ± 0.15b	8.85 ± 0.20b
VAN	26.21 ± 0.34b	25.91 ± 0.35b	24.44 ± 0.26b	27.54 ± 0.24a	29.34 ± 0.37a	29.01 ± 0.28a	28.92 ± 0.51a	28.90 ± 0.28a
CAF	28.56 ± 0.24a	23.88 ± 0.33c	26.73 ± 0.17b	27.97 ± 0.27a	30.22 ± 0.42c	29.65 ± 0.25d	31.18 ± 0.57b	32.44 ± 0.34a
PCO	22.01 ± 0.66a	21.58 ± 0.31a	22.18 ± 0.72a	23.09 ± 0.40a	22.52 ± 0.27b	23.00 ± 0.25a	23.64 ± 0.89a	23.67 ± 0.64a
FER	726.72 ± 28.82b	710.49 ± 7.13c	721.04 ± 4.89b	761.52 ± 17.30a	705.47 ± 17.83c	740.31 ± 8.18b	746.39 ± 9.74b	752.56 ± 16.55a
SIN	101.17 ± 0.65a	97.80 ± 1.27b	100.91 ± 1.09a	99.39 ± 0.89a	89.08 ± 0.78a	89.46 ± 1.10a	87.54± 0.73b	90.00 ± 0.60a
SAL	1.77 ± 0.07a	1.75 ± 0.06a	1.75 ± 0.05a	1.76 ± 0.05a	1.65 ± 0.01a	1.66 ± 0.01a	1.67 ± 0.00a	1.68 ± 0.01a
Total	936.32 ± 29.40b	911.30 ± 6.77c	926.97 ± 6.05b	972.67 ± 17.65a	916.80 ± 18.79c	952.12 ± 9.49b	959.09 ± 11.46b	968.69 ± 18.03a
Antiradical activity	0.210 ± 0.007b	0.204 ± 0.002c	0.203 ± 0.004c	0.218 ± 0.004a	0.205 ± 0.004c	0.214 ± 0.002b	0.214 ± 0.003b	0.217 ± 0.004a
**Phenolic acid**	**Rusałka**				**Wirtas**			
	**A**	**B**	**C**	**D**	**A**	**B**	**C**	**D**
PRO	3.23 ± 0.06b	3.26 ± 0.06a	3.32 ± 0.04a	3.37 ± 0.05a	3.31 ± 0.08d	5.60 ± 0.21b	4.49 ± 0.10c	7.33 ± 0.05a
POH	7.09 ± 0.16b	7.06 ± 0.17b	7.20 ± 0.12a	6.85 ± 0.26c	12.85 ± 0.48b	11.43 ± 0.27c	12.63 ± 0.20b	13.49 ± 0.12a
VAN	30.17 ± 0.26c	31.65 ± 0.35b	31.45 ± 0.25b	32.85 ± 0.42a	49.96 ± 1.46b	48.76 ± 1.18c	50.38 ± 0.74b	53.15 ± 0.48a
CAF	29.97 ± 0.75b	29.31 ± 0.34b	30.72 ± 0.22a	30.77 ± 1.03a	29.92 ± 0.41b	29.69± 0.78b	28.85 ± 0.26b	30.56 ± 0.33a
PCO	26.77 ± 0.50c	29.03 ± 0.59b	28.96 ± 0.43b	30.37 ± 0.79b	416.04 ± 5.71b	410.11 ± 9.93b	399.84 ± 1.08c	465.66 ± 2.58a
FER	782.68 ± 11.14b	787.97 ± 5.20b	805.06 ± 5.27a	806.02 ± 20.35a	977.87 ± 14.85a	866.91 ± 8.85c	885.55 ± 6.83b	954.76 ± 9.97a
SIN	90.16 ± 1.69b	94.82 ± 1.23a	91.37 ± 1.52b	88.05 ± 1.90c	64.37 ± 0.77a	55.55 ± 0.34b	63.51 ± 1.52a	57.35 ± 0.81b
SAL	1.74 ± 0.04c	1.75 ± 0.05c	1.77 ± 0.05b	1.79 ± 0.06a	2.82 ± 0.09b	2.81 ± 0.06b	2.85 ± 0.06b	3.11 ± 0.03a
Total	999.68 ± 13.73c	1013.51 ± 7.19b	1029.13 ± 7.71a	1029.76 ± 24.41a	1596.74 ± 20.89b	1470.89 ± 19.18c	1488.84 ± 9.32c	1628.98 ± 9.56a
Antiradical activity	0.227 ± 0.003b	0.227 ± 0.002b	0.225 ± 0.005b	0.231 ± 0.005a	0.357± 0.005a	0.329± 0.004b	0.332 ± 0.002b	0.365 ± 0.002a

Comparison of averages for combinations of cultivars (C) * silicon treatments (S) – S/C. Values are expressed as mean ± SD. Means in a row followed by different letters show significant differences ($p < 0.05$) according to the Tukey test.

Table S6. Influence of cultivar for alkylresorcinols content (µg/g of the grain) and antioxidant activity (in relation to *α*-tocopherol's activity = 1.00), in the study years (2019-2020).

Alkyl-resorcinol	2019				2020			
	**Harenda**	**Serenada**	**Rusałka**	**Wirtas**	**Harenda**	**Serenada**	**Rusałka**	**Wirtas**
C17:0	23.99 ± 0.58ab	21.80 ± 0.47bc	25.64 ± 0.53a	19.21 ± 0.39c	19.58 ± 0.91b	15.44 ± 0.77c	22.72 ± 1.04a	11.11 ± 0.49d
C19:1	40.12 ± 0.93b	33.48 ± 0.95c	47.07 ± 1.36a	29.15 ± 0.72d	17.94 ± 0.52ab	15.36 ± 0.82b	19.06 ± 1.01a	11.30 ± 0.89c
C21:1	30.68 ± 0.76b	30.84 ± 0.79b	33.98 ± 0.81a	29.47 ± 0.61b	47.21 ± 1.78bc	42.51 ± 2.51cd	57.70 ± 3.76a	51.92 ± 2.30ab
C21:0	276.21 ± 8.38b	222.76 ± 5.14c	315.48 ± 9.80a	188.48 ± 4.03d	173.77 ± 9.61a	91.54 ± 5.29b	164.05 ± 8.76ab	73.19 ± 4.44c
C23:0	55.91 ± 1.81b	46.49 ± 1.06c	60.86 ± 1.61a	41.94 ± 1.06c	25.08 ± 2.09a	11.92 ± 0.95b	22.92 ± 2.17a	9.73 ± 0.95c
C25:0	13.48 ± 0.43a	11.19 ± 0.17c	12.42 ± 0.38b	14.13 ± 0.28a	5.96 ± 0.58a	2.24 ± 0.19c	4.03 ± 0.33b	2.77 ± 0.30c
Total	618.18 ± 16.89b	517.88 ± 11.04c	692.40 ± 19.42a	437.50 ± 8.11d	431.29 ± 18.91a	284.29 ± 14.53b	448.23 ± 17.64a	240.90 ± 10.17b
Antiradical acitvity	0.219 ± 0.006b	0.182 ± 0.004c	0.244 ± 0.007a	0.155 ± 0.003d	0.152 ± 0.007a	0.100 ± 0.005b	0.158 ± 0.006a	0.093 ± 0.004b

Comparison of averages for combinations of years (Y) * cultivars (C) – C/Y. Values are expressed as mean ± SD. Means in a row followed by different letters show significant differences ($p < 0.05$) according to the Tukey test.

Table S7. Influence of silicon treatments (S) for alkylresorcinols content (µg/g of the grain) and antioxidant activity (in relation to *α*-tocopherol's activity = 1.00), in the study years (2019-2020).

Alkyl-Resorcinol	2019				2020			
	**A**	**B**	**C**	**D**	**A**	**B**	**C**	**D**
C17:0	22.30 ± 0.82b	22.17 ± 0.67b	23.86 ± 1.15a	22.31 ± 0.69b	18.80 ± 1.80a	16.57 ± 1.43b	16.82 ± 1.33b	16.67 ± 1.52b
C19:1	36.11 ± 2.04b	36.01 ± 1.72b	39.86 ± 2.80a	37.82 ± 2.25ab	16.80 ± 1.42a	15.15 ± 1.38b	15.75 ± 0.93b	15.96 ± 1.04b
C19:0	158.63 ± 9.72ab	153.54 ± 7.58b	170.74 ± 12.93a	158.28 ± 9.04ab	131.79 ± 12.71a	116.64 ± 9.32c	120.41 ± 7.95b	116.81 ± 9.97c
C21:0	250.89 ± 14.86ab	236.49 ± 12.47b	266.71 ± 21.38a	248.84 ± 14.04ab	144.32 ± 20.26a	123.38 ± 15.86ab	118.78 ± 10.26b	116.07 ± 10.26b
C23:0	51.98 ± 2.50ab	49.03 ± 1.96b	53.82 ± 3.50a	50.37 ± 2.22ab	21.51 ± 3.74a	16.95 ± 2.49ab	15.20 ± 1.59c	16.00 ± 1.48c
Total	563.70 ± 30.11ab	539.80 ± 24.11b	600.80 ± 42.73a	561.66 ± 27.80ab	390.23 ± 40.67a	338.75 ± 32.41b	337.77 ± 21.20b	337.97 ± 24.42b
Antiradical acitvity	0.199 ± 0.011ab	0.190± 0.008b	0.212 ± 0.015a	0.198 ± 0.010ab	0.138± 0.014a	0.119 ± 0.011b	0.121 ± 0.007b	0.126 ± 0.006ab

Comparison of averages for combinations of years (Y) * silicon treatments (S) – S/Y. Values are expressed as mean ± SD. Means in a row followed by different letters show significant differences ($p < 0.05$) according to the Tukey test.

Table S8. Influence of cultivars (C) and silicon treatments (S) for alkylresorcinol content (µg/g of the grain) and antioxidant activity (in relation to α-tocopherol's activity = 1.00) of spring wheat cultivars.

**Alkyl-resorcinol**	**Harenda**				**Serenada**			
	**A**	**B**	**C**	**D**	**A**	**B**	**C**	**D**
C17:0	24.16 ± 0.50a	22.42 ± 0.87b	20.92 ± 2.23c	19.63 ± 0.87cd	19.01 ± 1.68a	16.78 ± 1.47b	19.32 ± 1.79a	19.37 ± 1.60a
C19:1	30.22 ± 4.88a	29.01 ± 4.70b	29.82 ± 6.09ab	27.08 ± 4.38c	25.14 ± 3.73a	21.11 ± 3.73b	25.57 ± 4.84a	25.86 ± 4.28a
C19:0	177.84 ± 6.18a	156.06 ± 6.69b	158.97 ± 14.83b	146.19 ± 7.56c	129.25 ± 10.89b	116.17 ± 10.57c	135.53 ± 12.84a	132.25 ± 12.19a
C21:0	265.33 ± 21.34a	217.39 ± 18.24b	219.65 ± 32.86b	197.58 ± 21.30c	158.94 ± 26.89b	142.19 ± 31.11c	169.89 ± 32.31a	157.59 ± 29.51b
C23:0	49.97 ± 6.11a	37.12 ± 6.36bc	39.34 ± 8.78b	35.57 ± 6.59c	30.46 ± 7.07a	27.42 ± 8.67b	30.99 ± 8.41a	27.96 ± 7.09b
C25:0	12.47 1.51a	8.98 ± 1.64b	8.91 ± 1.91b	8.52 ± 1.73b	6.81 ± 1.96a	6.60 ± 2.30ab	6.86 ± 1.96a	6.59 ± 1.82ab
Total	601.99 ± 35.22a	510.40 ± 33.39b	514.49 ± 65.06b	472.05 ± 37.60c	407.65 ± 48.66b	361.04 ± 56.69c	427.51 ± 59.16a	408.15 ± 53.08b
Antiradical acitvity	0.213 ± 0.013a	0.180 ± 0.012b	0.182 ± 0.023b	0.167 ± 0.014c	0.144 ± 0.017b	0.126 ± 0.019c	0.151 ± 0.021a	0.144 ± 0.019b
**Alkyl-resorcinol**	**Rusałka**				**Wirtas**			
	**A**	**B**	**C**	**D**	**A**	**B**	**C**	**D**
C17:0	24.00 ± 1.64b	22.70 ± 1.04c	25.80 ± 1.40a	24.22 ± 0.97ab	15.01 ± 1.53a	15.58 ± 2.22a	15.32 ± 1.46a	14.74 ± 2.32b
C19:1	31.65 ± 5.58c	31.12 ± 5.48c	35.82 ± 7.38a	33.68 ± 7.16b	18.83 ± 3.56c	21.10 ± 5.08a	20.03 ± 3.59b	20.95 ± 4.18ab
C19:0	177.11 ± 11.41b	166.66 ± 8.17c	189.08 ± 15.48a	176.55 ± 11.03b	96.64 ± 6.66b	101.48 ± 9.98a	98.73 ± 4.73ab	95.17 ± 11.39b
C21:0	236.69 ± 25.65b	232.28 ± 26.28b	256.25 ± 47.76a	233.85 ± 40.52b	129.46 ± 27.63b	127.90 ± 26.52b	125.18 ± 21.86b	140.80 ± 29.06a
C23:0	41.43 ± 7.03b	42.19 ± 6.89ab	43.48 ± 11.41a	40.47 ± 9.60c	25.11 ± 7.98b	25.24 ± 6.96b	24.24 ± 6.41b	28.74 ± 7.80a
C25:0	8.17 ± 1.67ab	8.16 ± 1.59ab	8.93 ± 2.48a	7.65 ± 1.89b	7.98 ± 2.80b	8.38 ± 2.50b	8.16 ± 2.26b	9.27 ± 2.68a
Total	565.34 ± 43.36b	547.32 ± 43.71bc	604.01 ± 81.65a	564.58 ± 64.43b	332.87 ± 44.67b	338.34 ± 49.18b	331.13 ± 35.27b	354.46 ± 52.44a
Antiradical acitvity	0.199 ± 0.015ab	0.193 ± 0.015b	0.213 ± 0.029a	0.199 ± 0.023ab	0.117 ± 0.016bc	0.120 ± 0.018b	0.120 ± 0.011b	0.138 ± 0.011a

Comparison of averages for combinations of cultivars (C) * silicon treatments (S) – S/C. Values are expressed as mean ± SD. Means in a row followed by different letters show significant differences ($p < 0.05$) according to the Tukey test.

Figure of Contents:

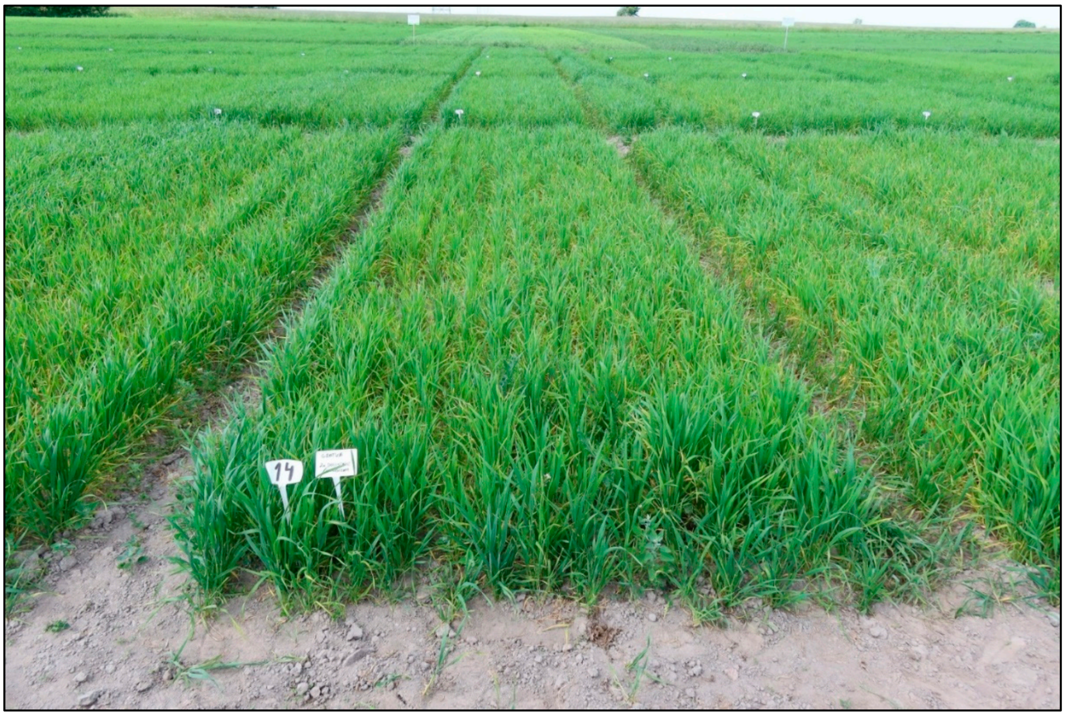

Figure S1. Field experiment on the effect of silica biopreparations on healthiness, yield and quality of spring wheat grain in Grabów (Poland).
